# Raman Spectroscopy Provides a Powerful Diagnostic Tool for Accurate Determination of Albumin Glycation

**DOI:** 10.1371/journal.pone.0032406

**Published:** 2012-02-29

**Authors:** Narahara Chari Dingari, Gary L. Horowitz, Jeon Woong Kang, Ramachandra R. Dasari, Ishan Barman

**Affiliations:** 1 Laser Biomedical Research Center, G. R. Harrison Spectroscopy Laboratory, Massachusetts Institute of Technology, Cambridge, Massachusetts, United States of America; 2 Division of Clinical Pathology, Beth Israel Deaconess Medical Center, Harvard Medical School, Boston, Massachusetts, United States of America; Tufts University, United States of America

## Abstract

We present the first demonstration of glycated albumin detection and quantification using Raman spectroscopy without the addition of reagents. Glycated albumin is an important marker for monitoring the long-term glycemic history of diabetics, especially as its concentrations, in contrast to glycated hemoglobin levels, are unaffected by changes in erythrocyte life times. Clinically, glycated albumin concentrations show a strong correlation with the development of serious diabetes complications including nephropathy and retinopathy. In this article, we propose and evaluate the efficacy of Raman spectroscopy for determination of this important analyte. By utilizing the pre-concentration obtained through drop-coating deposition, we show that glycation of albumin leads to subtle, but consistent, changes in vibrational features, which with the help of multivariate classification techniques can be used to discriminate glycated albumin from the unglycated variant with 100% accuracy. Moreover, we demonstrate that the calibration model developed on the glycated albumin spectral dataset shows high predictive power, even at substantially lower concentrations than those typically encountered in clinical practice. In fact, the limit of detection for glycated albumin measurements is calculated to be approximately four times lower than its minimum physiological concentration. Importantly, in relation to the existing detection methods for glycated albumin, the proposed method is also completely reagent-free, requires barely any sample preparation and has the potential for simultaneous determination of glycated hemoglobin levels as well. Given these key advantages, we believe that the proposed approach can provide a uniquely powerful tool for quantification of glycation status of proteins in biopharmaceutical development as well as for glycemic marker determination in routine clinical diagnostics in the future.

## Introduction

Glucose forms the most ubiquitous energy source in biology. In humans, glucose is primarily derived from the breakdown of carbohydrates in the diet or in body stores (glycogen), in addition to secondary endogenous synthesis from protein or from the glycerol moiety of triglycerides [1]. Importantly, even under diverse conditions (such as feeding, fasting and severe exercise), the blood glucose level is maintained within a fairly narrow interval, 70–120 mg/dL, by the homeostatic system of a healthy individual. This implies that, for an average person, the total quantity of glucose in the blood and body fluids is approximately 5 grams - a remarkably small number given the typical carbohydrate intake per day (*ca.* 150–200 grams). To maintain this natural balance, an intricate set of biomolecule interactions, modulated by glucoregulatory hormones such as insulin, needs to occur. However, in people afflicted with *diabetes mellitus*, the defective nature of carbohydrate metabolism (stemming from inadequate insulin production, response or both) leads to the presence of high blood glucose. Ominously, diabetes, which affects more than 25 million people in the US alone [2], has no established cure. As a consequence, early diagnosis and careful management of diabetes via frequent monitoring of glucose level is imperative in alleviating the severe associated health complications including micro- and macro-vascular diseases [3]. Presently, diagnosis and therapeutic monitoring of diabetes requires direct measurement of glucose by withdrawal of blood or/and interstitial fluid, both for clinical laboratory measurements as well as for self-monitoring of blood glucose. In order to reduce/eliminate the painful and invasive nature of these fingerprick measurements, minimally invasive [4]–[7] and non-invasive glucose monitoring has been actively pursued by a number of research laboratories [8]–[10] including our own [11]–[14].

In addition to these “gold standard” blood glucose measurements, measurement of glycated proteins has received considerable contemporary attention in the medical community for monitoring long-term glycemic control in diabetics [15], [16]. Specifically, glycated hemoglobin (HbA1c) and glycated albumin, which are formed by the non-enzymatic attachment of glucose to hemoglobin and serum albumin respectively, have been proposed as retrospective indices of the integrated blood glucose values over extended periods of time [17]–[20]. Importantly, these markers are not subject to the substantive variations observed in blood glucose concentration measurements, due to their intrinsic half-lives of 120 days (HbA1c) and 21 days (glycated albumin). While HbA1c measurements have been more extensively employed in clinical laboratories as an adjunct to blood glucose determinations, studies over the past decade have suggested that glycated albumin remains an “underestimated marker of diabetes” [21] and is, in fact, “a better indicator for glucose excursion than glycated hemoglobin in type 1 and type 2 diabetes” [22]. Notably, HbA1c values are significantly affected by shortening of erythrocyte life span [23]–[26] and are also prone to inaccuracies in the case of several chronic diabetes-related disorders (*e.g.* hemolytic or renal anemia and liver cirrhosis) [27]–[29]. On the other hand, glycated albumin is more sensitive to shorter term alterations in blood glucose values (due to its shorter half-life) and is not affected by changes in erythrocyte survival times nor by abnormal hemoglobin metabolism observed in some type 2 diabetes cases [28], [29]. From a clinical perspective, the value of glycated albumin determination has been further highlighted by reports of the strong correlation between glycated albumin concentrations and the development of serious diabetes complications including nephropathy [30], retinopathy [31] and arterial stiffening [32]. In light of these reports, one can reasonably infer that measurement of glycated albumin provides a crucial piece of information to complement plasma glucose and HbA1c determination for appropriate diabetes monitoring and therapeutics.

Current methods of glycated albumin determination include affinity chromatography, high-performance liquid chromatography and specific reagent-based colorimetric methods (*e.g.* thiobarbituric acid assay and nitro-blue tetrazolium assay). Nevertheless, as detailed by Sacks [33], these methods are not widely used because of their lack of suitability for routine clinical laboratory application. For example, the aforementioned colorimetric assays suffer from lack of specificity [34] and are vulnerable to the presence of free glucose, uric acid or lipemia [35]–[37]. Furthermore, the development of monoclonal antibodies specific to glycated albumin [38], while beneficial in principle, has not caused a rise in the availability of commercial glycated albumin assays at this present time.

In this context, optical/spectroscopic approaches provide a reagent-free detection method, which can be performed with little or no sample preparation. Previously, fluorescence spectroscopy has been employed to differentiate between glycated and unglycated albumin [39] as well as to characterize the effect of anti-oxidants on glycation-induced changes in proteins [40]. Nevertheless, to overcome the lack of specificity of intrinsic fluorescence spectroscopy (*i.e.* without addition of external dyes), investigators have used FTIR (Fourier-transform infrared) spectroscopy. FTIR spectroscopy is sensitive to changes in secondary structure and has provided valuable information on dynamic build-up of glycated albumin when incubating albumin with glucose [41], [42]. Alternately, one can obtain (complementary) vibrational information by using Raman spectroscopy. Notably, Raman can be used to analyze aqueous solutions as it does not suffer from the large water absorption effects associated with FTIR and, generally, provides higher spectral detail (due to less cluttering of peaks).

In this article, we propose a new Raman spectroscopy-based method for selective and sensitive determination of glycated albumin. Specifically, a derivative of spontaneous Raman spectroscopy, known as drop coating deposition Raman (DCDR) spectroscopy [43], is employed here to investigate the feasibility of reproducible identification and accurate quantitation of this important glycemic marker. In addition to the well-characterized advantages of Raman spectroscopy (such as excellent chemical specificity which obviates the need for exogenous reagents [44]), this technique provides signal amplification by pre-concentration of the analytes in solution. This pre-concentration – which is obtained by the simple drying of a droplet of the analyte solution (*i.e.* the so-called “coffee-ring effect”, stemming from the interplay of contact line pinning, solvent evaporation and capillary flow [45]) – enables the Raman measurement of analytes at 2–3 orders of magnitude lower than otherwise possible, without sacrificing their solution conformation [46]–[48].

Herein, our DCDR measurements reveal that glycation of albumin manifests itself in subtle but consistent changes in spectral features. In fact, in combination with standard multivariate chemometric methods, we observe that the glycated albumin can be discriminated from the unglycated samples with 100% accuracy. Moreover, we characterize the accuracy and precision of these measurements and demonstrate that the developed calibration models show high predictive power, even at substantially lower concentrations than typical physiological levels. Finally, we establish the limit of detection of this method for glycated albumin measurements. Based on the results obtained here, the proposed approach can be expeditiously employed for characterization of glycation status of proteins in mixtures, which is of critical importance because glycation can modify the stability, pharmacokinetics and immunogenicity of glycoprotein-based biopharmaceuticals. Additionally, Raman spectroscopy-based inspection of glycated albumin as a complementary glycemic marker exhibits substantive promise for similar determination in serum and whole blood samples. We envision that, in the future, this method will be enable us to provide real-time, reagent-free and simultaneous measurement of both glycated albumin and HbA1c providing a uniquely powerful tool for clinical laboratories.

## Materials and Methods

Our aim in this study is to develop DCDR as a complementary tool for qualitative and quantitative investigation of glycated albumin. As alluded to above, in this technique, spectroscopic measurements are performed on the coffee-ring pattern, where the analytes (*e.g.* proteins) are deposited from the drying drop. Herein, systematic experimental studies were initiated to achieve a two-fold objective. First, we assess the ability of the proposed approach, in conjunction with multivariate chemometric methods, to clearly distinguish pure albumin and glycated albumin samples. Second, we evaluate the quantitative ability of this method to precisely and accurately predict the concentration of glycated albumin at physiologically relevant levels and below. For the purpose of achieving these objectives, Raman spectroscopic measurements were performed on multiple drop-coated samples derived from a wide range of albumin and glycated albumin solutions, respectively. The acquired spectra were first examined for specific Raman bands and, subsequently, to discriminate between the samples. Subsequently, basic regression methodology was employed to quantitatively predict glycated albumin concentrations from the Raman spectroscopic measurements obtained from drop-coated depositions and to establish the prediction accuracy, precision and limit of detection of the proposed approach. In addition to the spectroscopic measurements, we also performed 2D spatial Raman mapping on representative drop-coated samples to investigate the uniformity (or the lack thereof) of the distribution of the analytes of interest (namely, albumin and glycated albumin). Evidently, the lack of substantial variance in measurements performed at a constant radial distance would manifest in higher reproducibility of the predicted concentrations from the drop-coated depositions.

### Experimental

For this study, a home-built Raman spectroscopic system equipped with a 785 nm CW Ti∶Sapphire laser (3900S, Spectra-Physics), which was pumped using a frequency-doubled Nd∶YAG laser (Millennia 5sJ, Spectra-Physics), was used. A liquid-nitrogen cooled CCD (LN/CCD 1340/400-EB, Roper Scientific) combined with a spectrograph (Kaiser Holospec f/1.8i) was used for collection of the spectra. A water immersion objective lens (Olympus UPLSAPO60XWIR 60X/1.20) focuses the laser to a spot size of approximately 1 µm on the sample and collects the backscattered Raman light. Due to the non-absorptive nature of the albumin and glycated albumin deposits, the power at the sample could be kept relatively high at *ca.* 30 mW without the possibility of optical and/or thermal damage to the samples. The detailed description of this system can be found in one of our laboratory's previous publications [49]. It should be noted that while this laboratory system was used in our experiments for the sake of convenience, a considerably simpler system comprising a single frequency diode laser would be adequate for these investigations.

**Figure 1:**
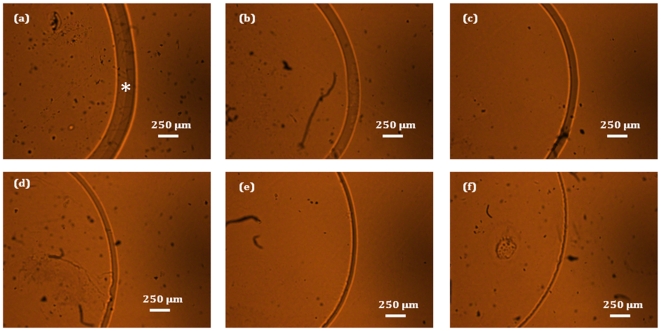
Bright field images of the drop-coated deposition rings. Bright field images of the drop-coated deposition rings obtained from air-drying of aq. glycated albumin samples. Evidently, the analytes are concentrated in the annular ring. The samples (a)–(f) are arranged in the order of descending concentration levels of glycated albumin, which is reflected in the widths of the corresponding rings. The asterisk in Fig. 1(a) represents the approximate center of the annular analyte-rich region. In all drop-coated deposition rings, spectroscopic measurements were performed at this approximate center location, where the Raman signal intensity was maximum.

Lyophilized powder samples of human serum albumin and glycated albumin were obtained from Sigma-Aldrich (St. Louis, MO, USA). The aqueous solutions of albumin were prepared in the range of 23–750 µM (the typical physiological range is between 3.5–5.5 g/dL or, *ca.* 510–710 µM [50]). Correspondingly, glycated albumin samples were formulated with concentrations in the range of 7–250 µM (typical physiological values are 10–25% of the above mentioned albumin concentrations [51]). All sample preparations are performed using high purity PESTANAL water (Fluka) to ensure the reproducibility of the measurements. Drop-coated depositions were prepared by pipetting aliquots (4 µL) of the prepared solutions on quartz coverslips (which were used to avoid the strong fluorescence interference of glass) and air-drying for approximately 20 minutes. The air-dried annular rings had widths in the range of 40–700 µm and scaled roughly in a linear fashion with respect to the concentrations (consequently, the albumin samples had larger annular ring widths in comparison to the glycated albumins samples). Figure 1 shows (portions of the) annular rings obtained from the glycated albumin samples after solvent evaporation, where (a) has the highest analyte concentration and (f) the lowest.

The acquisition time for all the Raman spectra was 10 seconds. For the classification and regression studies, spectra were collected from each sample at three different points at a constant radial distance from the center, with five replicate measurements at each location. The spectroscopic measurements were performed on the approximate center of the annular region (*e.g.* the position between the two concentric arcs marked by the asterisk in Fig. 1(a)) where the analytes accumulate due to solvent evaporation. It should be noted that this is different from the center of the entire ring, where little or no analyte deposition takes place (as confirmed by the lack of analyte-specific Raman spectral features in this region). Further mention of the drop-coated deposit should be taken as referring to the former, i.e. the analyte-rich annular region, unless otherwise noted. In addition for the investigation of the uniformity, 100 spectra were collected over a 80×80 µm field of view with 8 µm inter-point distance (2D spatial Raman mapping). The spectra acquired from these studies were subject to vertical binning and cosmic ray removal. No background correction was performed for the ensuing quantitative analysis due to the possibility of incorporation of spurious artifacts [52].

### Data Analysis

In order to investigate the classification ability of the proposed method between albumin and glycated albumin samples, we performed principal component analysis (PCA) (part of the Statistics Toolbox in MATLAB R2010b (MathWorks, Natick, MA)) on the entire dataset containing 180 spectra in all. In particular, 90 spectra were acquired from 6 concentrations of albumin and, similarly, 90 more spectra were collected from 6 glycated albumin concentrations (where the concentrations of each analyte were in the ranges mentioned previously). Principal component analysis (PCA) is a dimension reduction technique, which uses an orthogonal transformation to convert a set of observations of closely correlated variables into a set of values of uncorrelated variables called principal components (PC). The first few principal components (each PC is orthogonal to the preceding one) account for a high degree of the net variance and are often used for visualizing the primary differences between the classes [53], [54]. Here, logistic regression on the relevant principal components was pursued to obtain a separation plane between the samples and to ascertain the degree of classification accuracy. Logistic regression is a standard discriminate analysis technique, which is employed here to correlate the principal component scores with the sample classes (namely, albumin and glycated albumin) [55], [56].

Moreover, in order to illustrate the capability of DCDR to provide quantitative measurements of these analytes, partial least squares (PLS) regression was employed [57]. Specifically to gauge the reproducibility of the measurements in the 2D spatial Raman mapping study, a leave-one-sample-out PLS model (developed on the 75 spectra from 5 calibration samples) was used to predict the concentrations for the 100 prospective spectra collected over a 2D area of the ring on a representative glycated albumin sample.

For the quantification of the accuracy and precision of our measurements, we have performed leave-one-sample-out cross-validation procedure on the glycated albumin data acquired from the 6 samples. In the leave-one-sample-out cross-validation routine, one sample is left out when developing the calibration model and the resultant model is used to predict concentrations of the left out sample spectra. This procedure is repeated until all samples are left out and all concentrations are predicted. In particular, the calibration models are developed using 75 spectra (5 samples with 15 spectra per sample) and the predictions are performed on the remaining 15 spectra (1 sample) to obtain 15 predicted concentrations. This routine is repeated till all the glycated albumin samples (and spectra therein) are accounted for. Here, three figures of merit, namely relative error of prediction (REP), relative standard deviation (RSD) and limit of detection (LOD), were computed. The REP and RSD values correlate directly with the accuracy and precision of DCDR predictions, respectively. In the following, we provide the equations used for computing the figures of merit:

- Average relative error of prediction, REP:
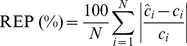
(1)where *N* is the number of spectra in the dataset, *c_i_* is the reference concentration and *ĉ_i_* is the predicted concentration.
- Average relative standard deviation of predicted concentrations, RSD:
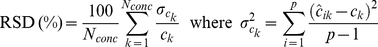
(2)where *N_conc_* is the number of distinct concentrations in the dataset, *p* is the number of spectra per concentration and *σ_ck_* is the standard deviation obtained at concentration *c_k_*.
- Limit of detection (LOD), as per the IUPAC definition [58], is computed from the best fit line obtained between predicted concentrations and reference concentrations [59]:
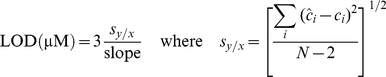
(3)where *s_y/x_* is the standard deviation of the residuals and is a measure of the average deviation of the predicted values from the regression line.

## Results and Discussion

**Figure 2:**
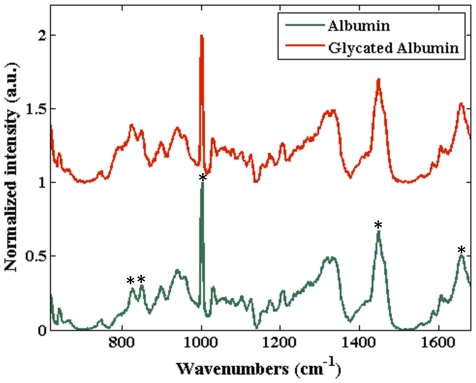
Raman spectra acquired from the drop-coated albumin and glycated albumin samples. Raman spectra acquired from the drop-coated albumin and glycated albumin samples derived from their corresponding aqueous solutions, respectively (the spectra are normalized and offset for the sake of clarity). The asterisks indicate the principal peaks, namely the 1655 cm^−1^ Amide-I band, the 1447 cm^−1^ CH_2_ deformation band, the 1002 cm^−1^ phenylalanine band and the tyrosine doublet at 828 and 850 cm^−1^.

Figure 2 shows Raman spectra acquired from typical drop-coated depositions of human serum albumin (green) and glycated albumin (red) solutions. For the sake of visual representation, the plots shown in Fig. 2 were subject to 5 spectra averaging from each sample and baseline-removal [60]. (It is worth emphasizing that the baseline-removed spectra were not used for any of the following analysis, as mentioned in Materials and Methods section.) The features observed in our (DCDR) albumin spectrum are consistent with those previously reported in the literature for albumin solutions [61]–[63]. A summary of the wavenumbers and their corresponding tentative Raman band assignments is given in Table 1. In particular, we note the presence of the following key features: 1655 cm^−1^ Amide-I band, 1447 cm^−1^ CH_2_ deformation band, 1002 cm^−1^ phenylalanine band and the tyrosine doublet at 828 and 850 cm^−1^. The Amide-I band is a characteristic feature of the α-helical (secondary) conformation of the polypeptide backbone stemming mainly from peptide C = O stretching vibration [64]. This is important because any change of this band would indicate a modification in the secondary structure of human serum albumin, which is predominantly an alpha-helical molecule (67%). Furthermore, the strong phenylalanine peak at 1002 cm^−1^ is reflective of the presence of 31 phenylalanine residues present in albumin (tryptophan may provide a small contribution to the intensity of the 1002 cm^−1^ band as well).

**Table 1:** Chemical assignments of vibrational modes for the Raman spectra acquired from drop-coated deposition of human serum albumin sample.

Wavenumber (cm^−1^)	Tentative Band Assignments
1655	Amide-I
1616	Tyr
1605	Phe
1584	Phe
1447	δ(CH_2_)
1335	δ(CH)
1319	δ(CH)
1208	Tyr+Phe
1172	Tyr
1157	υ(CN)
1125	υ(CN)
1102	υ(CN)
1089	υ(CN)
1031	Phe
1002	Phe
960	υ(CC)
940	υ(CCN)_sym_, υ(CC)
899	υ(CC)
850	Tyr
828	Tyr
667	υ(CS)
643	Tyr

Here, ν means stretching vibration; and δ deformation. Tyr, Trp and Phe refer to the tyrosine, tryptophan and phenylalanine residues, respectively.

Expectedly, the glycated albumin spectrum does not exhibit any gross differences in comparison to the albumin spectrum (although subtle changes in the Raman spectra in the 780–850 cm^−1^ region exist). Numerous studies have previously identified that (non-enzymatic) glycation of albumin occurs at multiple sites corresponding to the arginine, lysine and cysteine residues, which can be attributed to their high nucleophile properties [21],[65]–[67]. Since the Raman signature of albumin do not have significant contributions from these residues, one would anticipate that the corresponding glycation-induced changes would be subtle. Nevertheless, we hypothesize that these changes, although relatively small, are consistent and, as such, provide sufficient information to distinguish between albumin and glycated albumin samples. Specifically, such small changes are routinely detected using multivariate chemometric algorithms, which we have employed in the following analysis to test this hypothesis. It is also worth mentioning that glycation studies have indicated the conversion of albumin into a high β-sheet structure [68], [69] - another potential marker that may aid the classification of glycated and unglycated samples.

**Figure 3:**
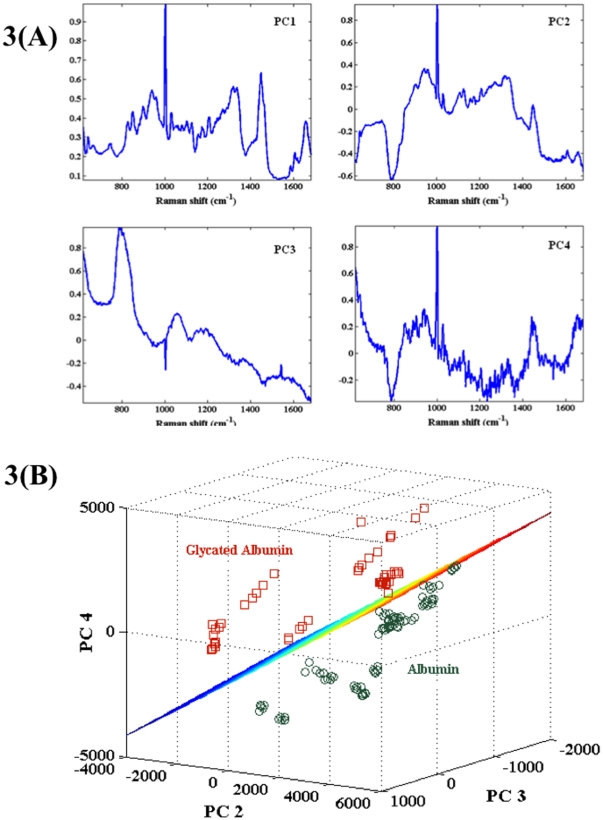
PCA decomposition of the spectral dataset. (A) The first four principal components corresponding to the entire spectral dataset acquired from the albumin and glycated albumin drop-coated deposition samples. These four principal components, combined, explain 99.74% of the net variance in the dataset. (B) Scores plot corresponding to principal components 2, 3 and 4 for the spectral dataset acquired from albumin and glycated albumin drop-coated rings. The albumin and glycated albumin samples are indicated by green circles and red squares, respectively. The optimal plane of separation, shown here, is constructed using a logistic regression algorithm (further details are noted in the text).

To this end, PCA was employed to visualize the underlying information from the multivariate spectral dataset, comprising both albumin and glycated albumin samples (90 spectra from 6 samples at different concentrations for each of the analytes). Figure 3(A) gives the first four principal components (which together account for 99.74% of the net variance). We observe that PC1 bears a striking resemblance to the pure albumin spectrum (and by extension to the glycated albumin spectrum, albeit to a somewhat lesser extent - especially in the 780–850 cm^−1^ region). PC2 retains some of the key features seen in PC1, although in different proportions. Interestingly, a new feature is observed at *ca.* 792 cm^−1^, which seems to stem from the differences in the aforementioned shoulder region in the tyrosine doublet between the glycated and unglycated samples. This feature is also present in a prominent manner in PC 3 and 4. In addition, these PCs have an interesting feature at *ca.* 1542 cm^−1^, which was not noted in the list of prominent bands in Table 1 and the origin of which is unclear at this present time.

The corresponding scores plot for PCs 2, 3 and 4 is given in Figure 3(B). (PC1 was excluded from this 3D plot because of its relatively lower discriminative power between the two sets of samples in comparison to the PCs employed here.) Remarkably, we can see a clear separation between the albumin and glycated albumin samples. To measure the discrimination ability of the proposed approach, we used logistic regression on the scores of PC 2, 3 and 4 (i.e. score_2_, score_3_ and score_4_, respectively). The optimal separation plane, based on these three parameters, was computed to be:
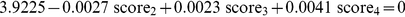
(4)This logistic regression algorithm gave a classification accuracy of 100%, as can be seen from Fig. 3(B). To test whether such a classification result could have been obtained from spurious correlations (such as system drift during measurements), we performed two control studies. First, we assigned the “albumin” and “glycated albumin” labels randomly to the 180 spectra, without any regard for their actual origin. We observed that the new “optimal” logistic regression algorithm barely gave 55% classification accuracy (which in this binary classification problem is akin to a random guess). This underlines the inability of the algorithm to predict the randomly assigned classes. Subsequently, we assigned class labels in correlation with the measurement order of the samples to investigate the possibility of temporal correlations (*e.g.* that stemming from system drift). In other words, we assigned the first 90 samples as albumin and the last 90 as glycated albumin (whereas the spectral measurements were performed in an arbitrary manner between the albumin and glycated albumin samples). Here, too, the “optimal” logistic regression algorithm displayed poor performance, and the overall classification accuracy was computed to be *ca.* 60%. Taken together, the actual logistic regression performance and the control studies validate our hypothesis that the chemometric methods can reliably predict class labels based on subtle, but consistent, differences in spectral features between albumin and glycated albumin samples. The control studies, in particular, also underline the robustness of DCDR in combination with multivariate classification to chance correlations.

**Figure 4:**
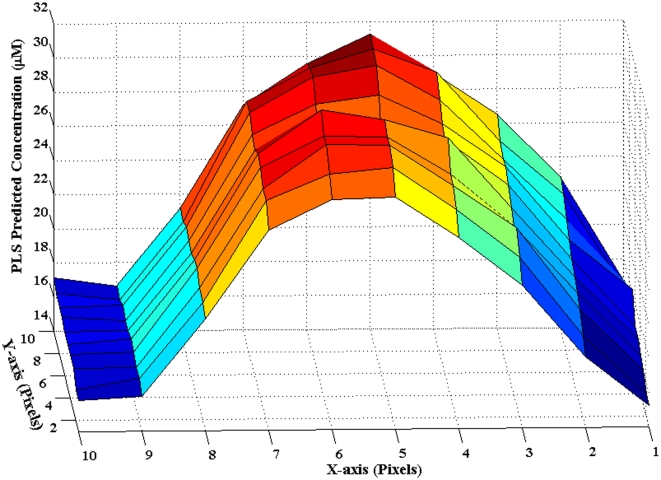
2D spatial Raman mapping of a glycated albumin drop-coated ring. 2D spatial Raman mapping based concentration prediction results for a representative glycated albumin drop-coated ring. The reference glycated albumin concentration in this sample is 31.25 µM. The field of view is 80×80 µm with a pixel-to-pixel distance of 8 µm. Here, the X- and Y-axis provide a close approximation to the radial and angular directions, respectively. Pixel 1 on the X-axis is located closer to the center of the ring (inner periphery) and pixel 10 is farthest away from the ring center.

Since PCA and logistic regression showed excellent discrimination ability from the DCDR spectra, we used a multivariate regression approach (PLS) to test the predictive power of the glycated albumin data. Before this test, however, it is important to characterize the reproducibility of the measurements by computing the potential variations in the radial and, more importantly, in the angular direction. Here, we have performed 2D spatial Raman mapping-based predictions on a representative glycated albumin sample (reference analyte concentration = 31.25 µM) using PLS calibration models developed on the other 5 sample spectra. Figure 4 plots the results of this analysis for the 100 spectra acquired over a 80×80 µm area of the annular ring. The profile along the radial direction (X-axis) shows an approximately symmetric shape with a steeper descending outer part (*i.e.* over pixels 8, 9 and 10) in comparison to the more gradual descent in the inner part of the ring (*i.e.* over pixels 3, 2 and 1). This is consistent with previous experimental observations of complete desiccation at the outer perimeter of the ring, primarily from oscillation of the droplet contact line [70]–[72]. On the other hand, there is a high degree of consistency between the predictions along the Y-axis, which for small distances (such as those considered here) provides a reasonable approximation for the angular direction. The coefficient of variation (i.e. the ratio of standard deviation to the mean of the predicted concentrations) along the Y-axis is calculated to be in the range of 0.014–0.074 with a mean of 0.038. This demonstrates the excellent reproducibility of the spectral predictions along the analyte-rich annular region of the ring, when the measurements are performed at a constant radial distance. Importantly, we also observe that the reference values of the glycated albumin concentrations are reproduced fairly accurately near the centre portion of the ring, i.e. the average of the predicted concentration over pixels 5 and 6 on the X-axis is 29.9 µM. Clearly, the absence of significant inhomogeneity in the drop-coated samples substantially increases the enthusiasm for systematic assessment of the prediction accuracy and precision across a wide range of concentrations.

**Figure 5:**
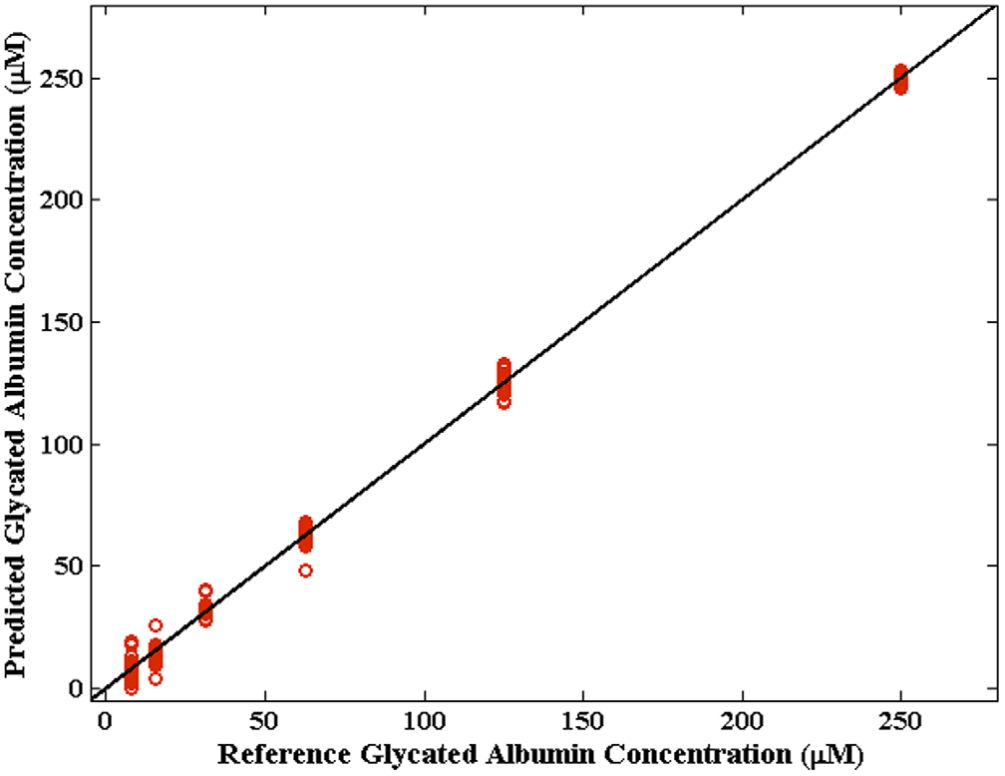
PLS prediction results of glycated albumin samples. Prediction results obtained using partial least squares (PLS) regression on glycated albumin samples. The solid line denotes y = x values.

Figure 5 shows the results of leave-one-sample-out cross-validation for the glycated albumin samples, where the reference and PLS predicted concentrations are given along the X- and Y-axis, respectively. The solid black line illustrates y = x and is given to explicitly understand the linearity of the response (or the lack thereof). From the figure, it is evident that the predicted values show excellent agreement with the reference concentrations and the corresponding correlation coefficient between these two set of values is calculated to be 0.9986. Further, the relative error of prediction (REP) was calculated to be *ca.* 16%, showing thereby that PLS provides very accurate predictions for the DCDR glycated albumin data over the entire concentration range of 7–250 µM. When the glycated albumin sample having 7 µM concentration is omitted from the dataset (as it is below the limit of detection of our system as discussed below), the REP value drops to 8.5%.

**Figure 6:**
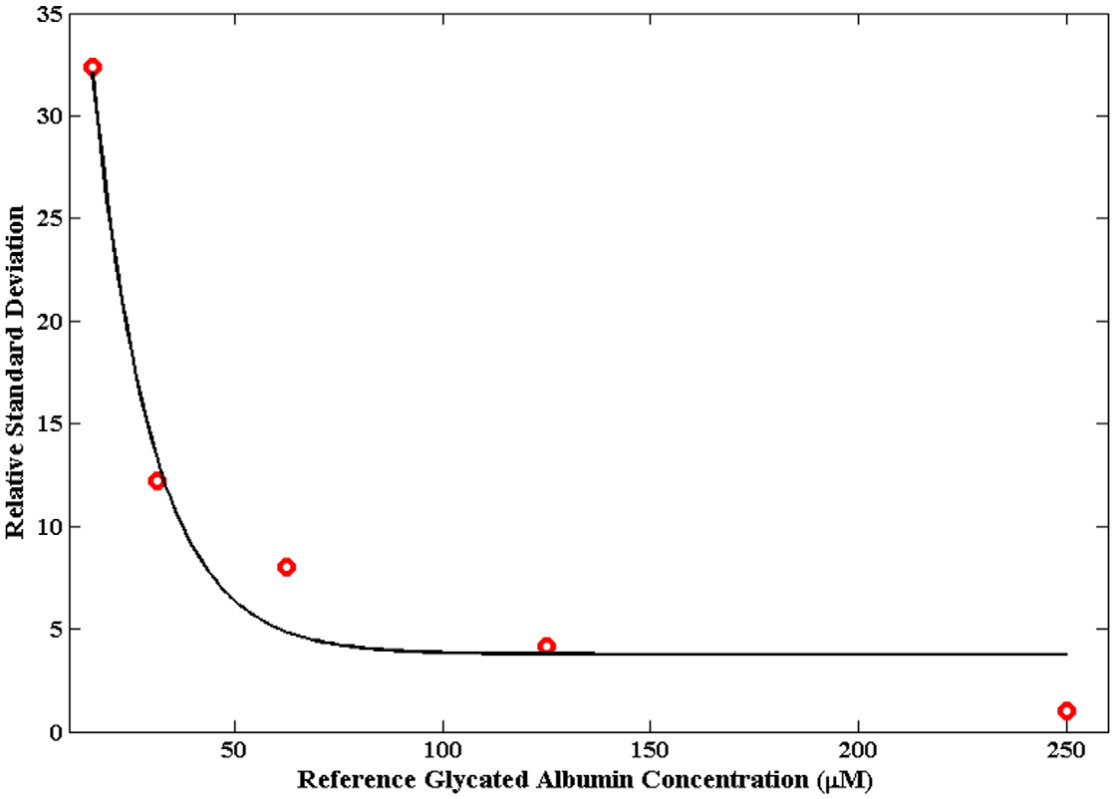
Relative standard deviation plot of precision for glycated albumin determination. Plot of precision as a function of reference glycated albumin concentration. The red circle gives the values computed from the experimental measurements and the solid black curve represents the best-fit exponential curve.

Finally, we evaluated the precision of our measurements using the relative standard deviation (RSD) metric. For the entire concentration range, our precision was observed to be 21.6%. Notably, when the 7 µM glycated albumin sample was not included in this analysis, the RSD metric reached a clinically acceptable value of 11.6%. Naturally, the precision gets worse as the concentration of the analyte decreases - a common characteristic of any spectrochemistry measurement. This aspect is revealed in Fig. 6 (also known as a precision profile in clinical chemistry), where the RSD is graphically plotted as a function of the reference glycated albumin concentration.

At this point, we determined the limit of detection (LOD) using the standard deviation of the residuals and the slope of the regression line, the so-called calibration plot method. Here, our LOD for glycated albumin was computed to be 13.7 µM, which is evidently higher than the lowest concentration used in this study (7 µM) but lower than the remaining sample concentrations. More importantly, this value is nearly 4 times less than the lowest physiological concentrations likely to be encountered in clinical settings (*ca.* 50 µM). Quantitatively speaking, the RSD is also 33% at the limit of detection (as per the IUPAC definition or 3σ detection limits) and therefore one can graphically extrapolate the RSD versus concentration plot to arrive at the LOD. Here, using this alternate method, we found the LOD value to be 14.7 µM. The small deviation from the previous value (13.7 µM) can be attributed to the deviation from an ideal exponential fit seen in Fig. 6. Nevertheless, it is reassuring that both methods generate very close numbers strengthening our confidence in the system's capability of measuring very low concentrations of glycated albumin.

In summary, a novel analytical procedure for reproducible identification and accurate quantification of glycated albumin has been proposed in this article. This method can also provide a real-time, reagent-free and largely non-perturbative alternative for probing glycation status of similar proteins in mixture solutions, which can aid in glycoprotein-based biopharmaceutical research and development. Moreover, while the experiments performed here establish the proof-of-principle for glycated albumin detection on the laboratory bench, further studies are currently underway to translate it to clinical settings. These studies involve the measurements on standard serum samples obtained from diabetic patients as well as normal human subjects (at the Beth Israel Deaconess Medical Center). Specifically, the samples are drawn by standard venipuncture into “serum separator” tubes, which contain a compound that speeds clotting and that, upon centrifugation, separates the serum from the cellular components of the blood (red blood cells, white blood cells, and platelets). Subsequently, drop coated plates are prepared (as in the aforementioned proof-of-concept studies) to test the efficacy of DCDR for glycated albumin determination in more physiologically relevant serum samples. (The results of this clinical study will be published in a follow-up article elsewhere.)

It is worth mentioning that in serum samples, the specificity (and the precision) of this method is unlikely to be significantly hampered due to two primary reasons. First, the concentrations of all other analytes (biomolecules) in serum are substantially lower in relation to physiological albumin concentration. In particular, albumin has a reference concentration range of 35–52 g/L, whereas the next highest concentration ranges belong to immunoglobulin G (7–16 g/L) and transferrin (2–3.6 g/L) (with almost all other serum constituents having concentrations less than 1 g/L [73]). Second, the strong and distinct Raman signal of albumin (and its glycated counterpart) does not show substantive spectral overlap with these major serum constituents [74], [75]. Finally, significant improvements to the current results can be made, especially through optimization of instrumentation and via enhanced chemometric modeling.

### Conclusions

This proof-of-concept study represents the first use of Raman spectroscopy, without application of extraneous reagents, to detect and quantify the concentration of glycated albumin, an important glycemic marker for long-term diabetes monitoring. Specifically, we have demonstrated that application of drop-coating deposition Raman spectroscopy can accurately discriminate glycated albumin from the unglycated variant, even at low µM concentrations. Further, in conjunction with standard multivariate analysis methods, we have shown that the limit of detection of the proposed approach for glycated albumin is nearly 4 times lower than the minimum physiological concentrations encountered in practice. The proposed method provides a promising alternative for glycated albumin determination as it is completely reagent-free and requires barely any sample preparation. The next step in translating this promising technology is to assess its predictive diagnostic value in multi-component mixtures, especially in serum samples. Additionally, in combination with recent studies of Raman-based characterization of protein glycosylation status [76], our investigations should advance the use of Raman and other spectroscopic modalities (such as fluorescence, FTIR and 2D-IR absorption spectroscopy [77]) for understanding the detailed structure and dynamics of albumin transformation caused by different analytes of interest, such as glucose and heavy metal ions [78].

Concomitantly, our laboratory is also engaged in investigating the clinical feasibility of HbA1c determination using DCDR. The combined determination of HbA1c and glycated albumin will provide a uniquely powerful metric in estimating the “true” glycemic history of a patient - a feature that is currently lacking in almost all clinical laboratories globally. The differences in the lifetime of these two important glycemic markers should shed interesting insight on the long-term glucose profile of a diabetic. Furthermore, the measurement of two markers may be imperative in certain clinical cases where one or the other may provide inaccurate estimates. For example, HbA1c values have been reported to underestimate the blood glucose levels in patients with hemolytic anemia [27], or those submitted to hemodialysis [79], whereas glycated albumin may not be an appropriate indicator for glucose excursion in pathologies that impact albumin metabolism, *e.g.* thyroid dysfunction and nephrotic syndrome [80], [81]. As a consequence, there is a significant clinical need for rapid and reliable glycemic history assessment that is (more) robust to other pathological changes. We believe this clinical need can be bridged by appropriate utilization of the proposed spectroscopic approach.
